# Reptiles in Guadeloupe (French West Indies) are a reservoir of major human *Salmonella enterica* serovars

**DOI:** 10.1371/journal.pone.0220145

**Published:** 2019-07-19

**Authors:** Stéphanie Guyomard-Rabenirina, François-Xavier Weill, Simon Le Hello, Sylvaine Bastian, Franck Berger, Séverine Ferdinand, Pierre Legreneur, Cécile Loraux, Edith Malpote, Blandine Muanza, Vincent Richard, Antoine Talarmin, Sébastien Breurec

**Affiliations:** 1 Unité Transmission, Réservoir et Diversité des Pathogènes, Institut Pasteur de Guadeloupe, Les Abymes, France; 2 Unité des Bactéries pathogènes entériques, Centre National de Référence des *Escherichia coli*, *Shigella* et *Salmonella*, Institut Pasteur, Paris, France; 3 Laboratoire de Microbiologie clinique et environnementale, Centre Hospitalier Universitaire de Pointe-à-Pitre/les Abymes, Pointe-à-Pitre, France; 4 Service de Santé des Armées, Centre d’épidémiologie et de santé publique des armées, Marseille, France; 5 INSERM, IRD, Sciences Economiques et Sociales de la Santé et Traitement de l’Information Médicale, Université d’Aix Marseille, Marseille, France; 6 CRIS EA 647, Université de Lyon, Villeurbanne, France; 7 Service de Pédiatrie, Centre Hospitalier Universitaire de Pointe-à-Pitre/les Abymes, Pointe-à-Pitre, France; 8 Institut Pasteur de Nouvelle-Calédonie, Nouméa, Nouvelle-Calédonie; 9 Faculté de Médecine Hyacinthe Bastaraud, Université des Antilles, Pointe-à-Pitre, France; Universidad de la Republica Uruguay, URUGUAY

## Abstract

The epidemiology of human *Salmonella enterica* infections in Guadeloupe (French West Indies) appears to be specific, with a higher prevalence of the subspecies *enterica* serovars Panama and Arechavaleta (Panama and Arechavaleta) than in other regions. A study was performed in Guadeloupe to identify the reservoir of *Salmonella* serovars by comparing their distribution in warm- and cold-blooded animals and in humans living in Guadeloupe and mainland France. Furthermore, a case–control study was conducted in 2012–2013 to identify the main epidemiologic risk factors for *S*. *enterica* infection among children under 15 years of age. Between June 2011 and December 2014, feces from 426 reptiles (322 anoles, 69 iguanas and 35 geckos) and 50 frogs distributed throughout Guadeloupe and nearby islands were investigated. The frequency of *S*. *enterica* carriage was 15.0% (n = 64) in reptiles but varied by species. The only significant risk factor for *S*. *enterica* infection was a more frequent presence of frogs in the houses of cases than in those of controls (*P* = 0.042); however, isolates were not collected. Panama and Arechavaleta were the two serovars most often recovered between 2005 and 2014 from humans living in Guadeloupe (24.5% (n = 174) and 11.5% (n = 82), respectively), which is in contrast to the low prevalence in mainland France (0.4%). Their presence at low frequencies in wild reptiles (4.6% (n = 3) and 3.1% (n = 2), respectively) and pigs (7.5% (n = 5) and 1.5% (n = 1), respectively) suggests a broad host range, and humans may be infected by indirect or direct contact with animals. These serovars are probably poorly adapted to humans and therefore cause more severe infections. The unusual subspecies *houtenae* serovar 43:_z4,z32_:- was a major subspecies in wild reptiles (24.6%, n = 16) and humans (9.4%, n = 67) but was not recovered from warm-blooded animals, suggesting that reptiles plays a key role in human infection.

## Introduction

All serovars of *Salmonella* belong to two species, *S*. *enterica* and *S*. *bongori*, although more than 99.5% of isolates are assigned to *S*. *enterica*. *S*. *enterica* comprises six subspecies: *enterica*, *salamae*, *arizonae*, *diarizonae*, *houtanae*, and *indica*. Most cases of human illness arise from *enterica* subspecies. Serovars Typhi, Paratyphi A, Paratyphi B and Paratyphi C are grouped as typhoidal *Salmonella*, and other serovars are described as non-typhoidal *Salmonella* (NTS). Typhoidal *Salmonella* are human host-restricted bacteria that cause typhoid and paratyphoid fever, which are systemic diseases, whereas a large number of NTS serotypes are generally responsible for acute gastroenteritis. The incidence of NTS infections is lower in industrialized than in developing countries, at about 45 per 100 000 inhabitants [1]. Invasive infections caused by NTS, such as meningitis and septicemia, can occur, particularly among young children, the elderly, malaria-infected and malnourished children, and immunocompromised people [2].

NTS can be host-generalists, capable of infecting or colonizing a broad range of vertebrate species, or host-specialists, adapted or restricted to particular non-human species [2]. In industrialized countries, NTS are transmitted to humans predominantly through the consumption of commercially produced food contaminated with livestock feces (e.g. meat, dairy products, poultry, and eggs) [2]. Outbreaks and sporadic cases of NTS have also been reported after direct or indirect contact with reptiles [3,4], as NTS are commonly found in the digestive tracts of reptiles (crocodiles, lizards, snakes, turtles) and amphibians (frogs, newts) [5–7]. Approximately 1.4 million human cases of *Salmonella* infection occur each year in the USA, of which about 74,000 are a result of exposure to reptiles and amphibians [8]. Within the European Union, it is estimated that less than 1% of cases of human salmonellosis are associated with exposure to reptiles. Reptile-related salmonellosis has been associated with young age, a high rate of hospitalization, and invasive disease [9,10]. Of the *Salmonella* serovars, 40% have been cultured predominantly from reptiles and are rarely found in other cold- and warm-blooded animals, suggesting that human infections with these serovars are of reptile origin.

Guadeloupe, a French overseas territory in the Caribbean, is a very high-resource country according to the Human Development Index in 2013. Although few data are available on the epidemiology of *Salmonella* in humans in the Caribbean, infections appear to be specific. In Guadeloupe, Panama and Arechavaleta were the most prevalent serovars recovered from 171 infants and children infected with *S*. *enterica* who were seen at the university hospital in Pointe-à-Pitre (Guadeloupe) between 2010 and 2014. The two serovars represented 50% of all *Salmonella* isolates in that study [11]. Surprisingly, they have been rarely encountered in mainland France or in other regions of the world [12]. In addition, these serovars are significantly associated with bacteremia (*P* < 0.001) [11]. Four cases of Panama meningitis were recently described in exclusively breastfed infants in French Guiana, suggesting a specific reservoir [13]. In Guadeloupe, wild reptiles and amphibians (e.g. anoles, geckos, iguanas and frogs) are commonly found in and around houses. We therefore conducted a study in Guadeloupe to identify the reservoir of Panama and Arechavaleta by comparing the distribution of *Salmonella* serovars in warm- and cold-blooded animals and in humans. A matched case–control study was also conducted to determine the main epidemiologic risk factors among children with *S*. *enterica* infection.

## Material and methods

### *S*. *enterica* isolates from human samples

*S*. *enterica* clinical isolates were received for serotyping between January 2004 and December 2014 by the French national reference center for *Escherichia coli*, *Shigella* and *Salmonella* (FNRC-ESS) (Institut Pasteur, Paris, France) from public and private clinical laboratories in Guadeloupe and mainland France. If more than one isolate with the same serovar was recovered from the same patient, only the first was included. Epidemiologic data (date and site of isolation, age and gender of the patient, history of travel abroad) were recorded when available.

### *S*. *enterica* isolates from warm-blooded animals

*Salmonella* spp. were isolated from samples collected at poultry farms (droppings, dust, eggs, and poultry meat) and pig and beef farms (feces, carcasses, and meat) in Guadeloupe during 2010–2014 for sanitary inspections. Serotyping was performed at the Institut Pasteur of Guadeloupe, at the FNRC-ESS and at the French Agency for Food, Environmental and Occupational Health and Safety (ANSES). Data were compiled from the different databases.

#### *S*. *enterica* isolates from wild cold-blooded animals

Between June 2011 and December 2014, a single cloacal swab was taken from 322 endemic anoles of 3 species and 11 sub-species at 85 sampling sites distributed throughout Guadeloupe and nearby islands (Les Saintes, Marie-Galante, La Désirade, and Petite-Terre) (S1 Table). Feces from iguanas living in colonies were collected at 10 sampling sites: 45 from the endemic *Iguana delicatissima* and 24 from the invasive *I*. *iguana* (S1 Table). Fifty frogs and 35 geckos at 8 and 12 sampling sites, respectively, were trapped and placed in sterile vials (S1 Table). Fecal droppings were collected within 24 h of capture. After sampling, all frogs and lizards were released at the capture sites. All samples were placed at +4°C immediately after sampling and were processed within 4 h.

All procedures were approved by the regional environment, planning and housing agencies and by the Guadeloupe National Park. The project was also approved by the Committee for Ethics in animal experiments of the French West Indies and Guyana (reference 69-2012-4). The care and use of animals were performed accordingly with the French Decree No 2013–118 of 1 February 2013 on the protection of animals, which meets the European Union Directive 2010/63 on the protection of animals used for experimental and other scientific purposes.

Samples were incubated in 9 ml of buffered peptone water for 16–20 h at 37°C. Three drops (75μl) of pre-enrichment broth were inoculated onto modified semi-solid Rappaport-Vassiliadis (MSRV) agar and incubated for a maximum of 48 h. Positive MSRV spots were streaked onto a specific medium, xylose–lysine deoxycholate (XLD) agar, and incubated for up to 48 h. Presumptive *Salmonella* colonies (H2S positive) on XLD agar were isolated and identified on API 20E test strips (bioMérieux, Marcy L'Etoile, France). Serotyping was performed on five colonies from each sample.

### Serotyping of *S*. *enterica* isolates

Isolates were serotyped on the basis of somatic O and both phase 1 and phase 2 flagellar antigens in agglutination tests with antisera (Bio-Rad, Marnes-La-Coquette, France) as specified in the White-Kauffmann-Le Minor scheme [14].

### Epidemiological study

A case–control study was carried out in 2012–2013. Cases were patients admitted to the pediatric department of the university hospital of Pointe-à-Pitre with an *S*. *enterica* infection (acute gastroenteritis or bacteremia). At the inclusion of each case, a trained scientist administered a standardized questionnaire by telephone with the parents to collect demographic, environmental and lifestyle information on the *S*. *enterica* infection; the same person selected age-matched (± 5 years) children without *S*. *enterica* infection or carriage and administered the same questionnaire to the parents.

The study protocol was approved by the French Advisory Committee on Information Processing in Material Research in the Field of Health (CCTIRS 11–40). Written informed consent to participate in the study was obtained from the parents of all children included in the case–control study.

### Data analysis

Statistical analyses were performed with R software [15]. Conditional tests were used because assumptions of traditional parametric tests were not met, with small samples and non-normal distributions. Thus, resampling procedures were implemented with the “coin” package, which does not assume random sampling from well-defined populations. Resampling provides especially clear advantages when assumptions of traditional parametric tests are not met. In a two-way contingency table, inference was based on 9999 Monte-Carlo resampling. Statistical differences were considered significant for two-sided *P* values < 0.05.

## Results

### *Salmonella* species, subspecies and serovars in humans in Guadeloupe

A total of 710 *S*. *enterica* isolates were collected between 2005 and 2014. Four subspecies were recovered: *enterica* (n = 669, 94.2%), *houtenae* (n = 39, 5.5%), *salamae* (n = 1), and *diarizonae* (n = 1). Of the 68 serovars found, the most prevalent were Panama (24.5%, 174/710), Arechavaleta (11.5%, 82/710), Enteritidis (9.4%, 67/710), 4,[5],12:i:- (monophasic variant of Typhimurium) (9.4%, 67/710), and Newport (7.2%, 51/710) (Table 1). Panama was the most frequently isolated serovar every year, except in 2011 and 2014. Arechavaleta ranked in the top five isolated serovars each year, except in 2006 and 2010. During the study period, one isolate of serovar Paratyphi B and 13 isolates (1.8%) of Typhi were isolated (Table 1).

**Table 1:** Distribution of the 10 most frequent *Salmonella* serovars in humans in Guadeloupe and mainland France between 2005 and 2014.

	Guadeloupe											Mainland France
Rank	2005	2006	2007	2008	2009	2010	2011	2012	2013	2014	2005–2014	2005–2014
	n = 73	n = 39	n = 48	n = 32	n = 79	n = 121	n = 73	n = 85	n = 101	n = 59	n = 710	n = 87 305
1	Panama (25%)	Panama (23.1%)	Panama (33.3%)	Panama (34.4%)	Panama (20.2%)	Panama (24%)	4,[5],12:i:- (20.5%)	Panama (27%)	Panama (30.7%)	Enteritidis (22%)	Panama (24.5%)	Typhimurium (40.5%)
2	Enteritidis (14%)	43:z_4_,z_32_:- ^a^ (12.8%)	Arechavaleta (18.7%)	Arechavaleta (25%)	4,[5],12:i:- (16.5%)	Newport (13.2%)	Newport (11%)	4,[5],12:i:- (15.3%)	Arechavaleta (12.9%)	Panama (22%)	Arechavaleta (11.5%)	Enteritidis (9.6%)
3	Newport (8.2%)	Enteritidis (10.3%)	43:z4,z32:-:-^a^ (12.5%)	43:z_4_,z_32_:- ^a^ (6.2%)	Arechavaleta (10.1%)	4,[5],12:i:- (10.7%)	Panama (11%)	Arechavaleta (14.1%)	Enteritidis (9.9%)	Arechavaleta (20.3%)	Enteritidis (9.4%)	4,[5],12:i:- (9.4%)
4	Arechavaleta (5.5%)	Typhimurium (7.7%)	Enteritidis (8.3%)	4,[5],12:i:- (6.2%)	Infantis (7.6%)	Typhimurium (9.9%)	Arechavaleta (11%)	Enteritidis (9.4%)	Newport (8.9%)	4,[5],12:i:- (6.8%)	4,[5],12:i:- (9.4%)	Infantis (2.0%)
5	Typhimurium (4.1%)	Infantis (7.7%)	Typhimurium (6.2%)	Typhi (6.2%)	Enteritidis (6.3%)	Infantis (8.3%)	Infantis (5.5%)	43:z_4_,z_32_:- ^a^ (9.4%)	4,[5],12:i:- (6.9%)	Newport (6.8%)	Newport (7.2%)	Typhi (1.7%)
6	Agona (2.7%)	Manhattan (5.1%)	Newport (6.2%)	48:g,z_51_:- ^a^ (3.1%)	Indiana (6.3%)	Arechavaleta (6.6%)	Enteritidis (5.5%)	Rubislaw (5.9%)	Infantis (4.9%)	Rubislaw (6.8%)	Infantis (5.3%)	Virchow (1.6%)
7	Infantis (2.7%)	Mississippi (5.1%)	Infantis (2.1%)	Bredeney (3.1%)	Rubislaw (5.1%)	Enteritidis (6.6%)	Rubislaw (4.1%)	Infantis (3.5%)	Braenderup (4.9%)	Infantis (5%)	43:z_4_,z_32_:- ^a^ (4.9%)	Newport (1.6%)
8	Kottbus (2.7%)	Rubislaw (5.1%)	Braenderup (2.1%)	Infantis (3.1%)	Typhi (5.1%)	Rubislaw (5.8%)	Typhi (4.1%)	Newport (3.5%)	Rubislaw (3.9%)	43:z_4_,z_32_:- ^a^ (3.4%)	Rubislaw (4.4%)	Derby (1.6%)
9	Manhattan (2.7%)	50:g,z_51_:-^a^ (2.6%)	Derby (2.1%)	Manhattan (3.1%)	43:z_4_,z_32_:- ^a^ (3.8%)	43:z_4_,z_32_:- ^a^ (4.1%)	Weltevreden (3.7%)	Oranienburg (3.5%)	Typhimurium (2%)	Javiana (1.7%)	Typhimurium (4%)	Kentucky (1.5%)
10	Rubislaw (2.7%)	6,8:e,h:- (2.6%)	Javiana (2.1%)	Oranienburg (3.1%)	Newport (2.5%)	Agona (1.6%)	Typhimurium (2.7%)	Aberdeen (1.2%)	Uganda (2%)	Miami (1.7%)	Typhi (1.8%)	Hadar (0.8%)

^a^ *S*. *enterica* subsp. *houtenae*

*Salmonella* isolates were recovered mainly from stool (82.1%, 583/710) and blood (12.7%, 90/710) samples. Of the blood isolates, 79 were NTS, of which 48 (60.7%) were Panama (n = 26, 32.9%) or Arechavaleta (n = 22, 27.8%) serovars.

### *Salmonella* species, subspecies, and serovars in humans in mainland France

A total of 87 305 *Salmonella* isolates from humans were investigated between 2005 and 2014. The most prevalent serovars were Typhimurium (40.5%), Enteritidis (9.6%), and 4,[5],12:i:- (9.4%) (Table 1). Panama was recovered only rarely (n = 349, 0.4%).

For the 180 Panama isolates collected between 2010 and 2014, information on travel abroad was available for 61 (33.9%) patients: 54 (88.5%) had traveled in the French West Indies or in South or Central America, and 7 (5.6%) had no history of travel.

### *Salmonella* species, subspecies, and serovars in livestock in Guadeloupe

During the period 2010–2014, 386 *S*. *enterica* isolates were recovered: 319 (82.6%) from poultry, 60 from pigs (15.5%), and 7 from cattle (n = 7) (Table 2). A total of 37 serovars were found; Newport (20.7%, 80/386), 4,[5],12:i:- (10.1%, 39/386), and Havana (8.8%, 34/386) were the most frequent. Newport (25.1%, 80/319) predominated in poultry, whereas 4,[5],12:i:- predominated in pigs and cattle (31.3%, 21/67). Panama and Arechavaleta were recovered only from pigs and at lower frequency, 7.5% (n = 5) and 1.5% (n = 1), respectively.

**Table 2:** Distribution of the 10 most frequent *Salmonella* serovars in cold- and warm-blooded animals in Guadeloupe.

Rank	Cold-blooded animals									Warm-blooded animals								
	Anolis	n	(%)	Iguanas	n	%	Total	n	%	Poultry	n	%	Pigs and cattle	n	%	Total	n	%
1	43:z_4_.z_32_:-^a^	16	44.4	Schwarzengrund	20	71.4	Schwarzengrund	20	30.8	Newport	80	25.1	4,[5],12:i:-	21	31.3	Newport	80	20.7
2	Pomona	9	25	Infantis	4	14.3	43:z_4_.z_32_:-^a^	16	24.6	Havana	34	10.7	London	21	31.3	4,[5],12:i:-	39	10.1
3	Rubislaw	4	11.1	Panama	2	7.1	Pomona	9	13.8	Indiana	33	10.3	Uganda	7	10.4	Havana	34	8.8
4	Newport	3	8.3	Newport	1	3.6	Infantis	5	7.7	Infantis	30	9.4	Derby	6	9	Indiana	33	8.5
5	Arechavelata	2	5.5	Lesabymes (67:d:1,7)	1	3.6	Newport	4	6.1	4,[5],12:i:-	18	5.6	Panama	5	7.5	Infantis	30	7.8
6	Infantis	1	2.7				Rubislaw	4	6.1	Albany	15	4.7	Agona	2	3	London	22	5.7
7	1,44:-:- ^a^	1	2.7				Panama	3	4.6	Uganda	15	4.7	Albany	1	1.5	Uganda	22	5.7
8	Panama	1	2.7				Arechavelata	2	3.1	Cerro	12	3.8	Anatum	1	1.5	Albany	16	4.1
9							1.44:-:- ^a^	1	1.5	Enteritidis	10	3.1	Arechavaleta	1	1.5	Cerro	12	3.1
10							Lesabymes (67:d:1,7)	1	1.5	Typhimurium	8	2.5	Enteritidis	1	1.5	Enteritidis	11	2.8

^a^ *S*. *enterica* subsp. *houtenae*

### *Salmonella* species, sub-species, and serovars in cold-blooded animals in Guadeloupe

The frequency of *S*. *enterica* carriage in cloacal specimens from the 426 wild reptiles investigated was 15% (n = 64) but varied by species, from 0 in geckos (0/35), 11.2% (36/322) in anoles, to 40.5% (28/69) in iguanas (Table 3). No isolates were collected from frogs (0/50).

**Table 3:** Risk factors for *Salmonella enterica* infection.

Risk factor	Cases (N = 50)	Controls (N = 25)	Univariate analysis		
			*P*	Crude OR	(95% CI)
Age months, mean (standard deviation)	30.7 (27.8)	39.4 (33.4)	0.301		
Male sex	31 (62.0)	14 (56.0)	0.627	1.3	(0.4–3.8)
Way of life					
Live in the countryside	35 (70.0)	14 (56.0)	0.304	1.8	(0.6–5.5)
Live in an individual house	36 (72.0)	17 (68.0)	0.790	1.2	(0.4–3.8)
Live in a house with a garden	29 (58.0)	15 (60.0)	1.000	0.9	(0.3–2.7)
Presence of reptiles					
In the garden	30 (60.0)	14 (56.0)	0.806	1.2	(0.4–3.5)
In the house	36 (72.0)	15 (60.0)	0.307	1.7	(0.5–5.3)
Presence of amphibians					
In the garden	26 (52.0)	10 (40.0)	0.462	1.6	(0.6–4.9)
In the house	23 (46.0)	5 (20.0)	0.042	3.4	(1.0–13.8)
Presence of pets					
Dogs	8 (16.0)	4 (16.0)	1.000	1.0	(0.2–5.1)
Cats	9 (18.0)	6 (24.0)	0.553	0.7	(0.2–2.7)
Systematic handwashing before meals	31 (62.0)	19 (76.0)	0.301	1.9	(0.6–7.0)
Consumption of garden vegetables or fruits	27 (54.0)	11 (44.0)	0.469	1.5	(0.5–4.4)

All the isolates belonged to the *enterica* species. Two subspecies were recovered: *enterica* (n = 48, 73.8%) and *houtenae* (n = 17, 26.2%). Ten serovars were found, eight in anoles and five in iguanas. The three most prevalent were Schwarzengrund (30.8%, 20/65), 43_:z4,z32:-_ (*houtenae* subspecies) (24.6%, 16/65), and Pomona (13.8%, 9/65) (Table 3). *S*. 43:z_4_,z_32_:- predominated in anoles (44.4%, 16/37), whereas Schwarzengrund predominated in iguanas (71.4%, 20/28). Panama and Arechavaleta were found in 4.6% (n = 3) and 3.1% (n = 2) of specimens, respectively (Table 2).

Serotyping was performed on five colonies from each reptile, but only one anole contained two different serovars: one belonging to Newport and one to 43:z_4_,z_32_:- (*houtenae* subspecies).

Significant associations between prevalent serovars and location were found only for Pomona and Schwarzengrund. Pomona was found exclusively in anoles sampled on two islands (Marie-Galante and La Desirade), and Schwarzengrund was isolated from endemic iguanas on two islands (Petite-Terre and La Désirade) (83.3%, 20/24) but not in invasive iguanas sampled on the main island.

### Epidemiological study

A total of 75 children were enrolled in the study (50 cases and 25 controls); 45 were boys. The mean age at inclusion was 33.2 months, and the age distribution was: 19 (25.3%) aged 0–11 months, 22 (29.3%) aged 12–23 months, 20 (26.7%) aged 24–59 months, and 14 (18.7%) aged 60–126 months.

Demographics, environmental characteristics and lifestyle information are summarized in Table 3. No significant difference was found between cases and controls in environment or lifestyle factors, except for a more frequent presence of amphibians in the houses of cases than in those of controls (*P* = 0.042) (Table 3).

## Discussion

The two most frequent *Salmonella* serovars in humans living in Guadeloupe were Panama and Arechavaleta. Panama was also the most prevalent serovar recovered from humans in two French overseas territories, Martinique in the Caribbean (35% of all isolates investigated between 1990 and 1994) and French Guiana in South America (11.7% in 2011) [12,16]. Although few data are available on the epidemiology of *Salmonella* infections in humans in this region, the Panama serovar appears to be highly prevalent. It was also the most prevalent serovar in humans in Colombia and Chile [17,18]. The prevalence is higher than those in other regions of the world, including mainland France, where it was found only rarely (0.4% of all isolates investigated, most collected from patients with a history of travel to either the French West Indies or South or Central America). To the best of our knowledge, the only cases of human infection with Arechavaleta have been reported in New Zealand [19], but at a lower frequency than in Guadeloupe, with nine cases reported between 1997 and 2016. As in mainland France and more generally in Europe and other parts of the world, the monophasic variant of the serovar Typhimurium (4,[5],12:i:-) has predominated in Guadeloupe since the mid-2000s [20,21]. Nevertheless, although Typhimurium remains the most frequent serovar in mainland France, 4,[5],12:i:- replaced its biphasic variant in Guadeloupe after 2008. Unsurprisingly, 4,[5],12:i:- was the second most frequent serovar isolated from livestock in Guadeloupe during the study period, supporting the role of pigs and poultry in its transmission to humans [22]. The other major serovars found in humans are commonly associated with human infections, except for 43:z_4_,z_32_:- (*houtenae* subspecies). To our knowledge, this serovar was recovered in one case of osteomyelitis in a Taylor’s cantil pit viper but has not been isolated in humans[23]. As these unusual serovars were not (43:z_4_,z_32_:-) or rarely (Panama and Arechavaleta) found in the warm-blooded animals sampled, we investigated a reptilian source of contamination in order to identify their reservoir.

The overall frequency of *S*. *enterica* carriage in the reptiles studied in Guadeloupe was 15%, which is in the lower range of the reported values (13–57%). *Salmonella* carriage rates differed by species in our study: no isolates were recovered from geckos, in agreement with the low prevalence reported in most studies [24,25]. The rate in anoles was 11.4%, between the two reported values (0 and 33%) [5,26], and *Salmonella* was found in 40.5% of iguanas tested, also in the middle of the range of reported values (26–98%) [12,27–29]. Several factors might explain the variation among studies in the recovery rates in reptiles. The frequency of carriage might differ by species, and each group is composed of several species. Carriage frequency also differed by habitat. Crowding of reptiles favors the transmission of NTS, as seen in the high frequency of Schwarzengrund in iguanas from the small islands Petite Terre (1.68 km^2^) and La Desirade (21.42 km^2^). In addition, reptiles are intermittent *Salmonella* shedders [30,31]. In most studies, including ours, specimens were taken only once, which results in underestimates of the rate of *Salmonella* carriage. Lack of a standard method for *Salmonella* isolation and differences in the sampling technique (cloacal swabbing of protected animals versus fresh fecal samples from sacrificed animals in other studies) are possible explanations [32].

The case–control study of the main epidemiologic risk factors of *Salmonella* infection showed, despite the small sample, that the presence of frogs in homes was significantly associated with [24,25] *Salmonella* infection. However, we were unable to isolate *Salmonella* from 50 frogs, perhaps because of selection bias, as we could not sample frogs from the houses of cases. Furthermore, an observer bias is possible since frogs are more easily observed in houses than anoles or geckos. Previous studies reported very low rates of carriage in frogs [24,33].

In contrast to the rates observed in other studies (< 50%), in our study most of the isolates recovered from reptiles were assigned to subspecies *enterica* (69%), which is commonly isolated from warm-blooded animals. Although the subspecies *salamae*, *arizonae*, *diarizonae* and *houtanae* are known to be harbored by reptiles, we recovered only *houtenae*.

The subspecies *houtenae* serovar 43:z_4_,z_32_:- was prevalent in reptiles and humans in Guadeloupe but was not found in livestock, suggesting a strict reptilian origin of human infections caused by this uncommon serovar. The presence of Arechavaleta and Panama in anoles and iguanas in Guadeloupe also indicates a reptilian reservoir for both serovars. This is not surprising, as Panama has previously been isolated from frogs, toads, turtles, lizards, and snakes [12,34,35] and Arechavaleta from cane toads [36]. However, their host range is certainly much larger, as shown by its presence in pigs in Guadeloupe. Both serovars were also found in previous studies in warm-blooded animals; Panama was found in wild birds, pigs, poultry, and Indian mongooses [37–41] and Arechavaleta in dogs and Indian mongooses [36,39,42]. In Guadeloupe, wild reptiles and amphibians (e.g. anoles, geckos, iguanas and frogs) are commonly found in and around houses. Guadeloupe is also a highly anthropized island, suggesting that close promiscuity between warm- and cold-blooded animals is at the origin of inter-species transmission. Therefore, we hypothesize that Panama and Arechavaleta are transmitted to humans either by direct contact with animals, in particular reptiles, or indirectly, through the consumption of food contaminated with livestock or reptile feces.

The low prevalence of serovars Panama and Arechavaleta in cold- and warm-blooded animals contrasts with that observed in human *Salmonella* infections. Evolutionary models suggest that host-adapted *Salmonella* serovars, such as serovars Typhi and Paratyphi A in humans, tend to be of high virulence, causing higher mortality rates than those with a broad host range, such as serovars Typhimurium and Enteritidis [43]. These host-adapted *Salmonella* serovars can cause illness in all age groups, whereas those with a broad host range tend to be more frequently associated with disease in young animals than in adults, suggesting that they are not optimally adapted to cope with a fully mature immune system [43]. Finally, chronic carriage, which develops more frequently following systemic infections by that host-adapted *Salmonella* serovars increases transmissibility [43]. Serovars Panama and Arechavalata are lesser virulent than serovars Typhi and Paratyphi A, as illustrated by the fact that they cause lesser mortality rates (0 to 13% versus 12 to 32%) and that they cause disease primarily opportunistically [11,43–46]. The number of bacteremia cases associated with serovars Panama and Arechavalata were higher in infants and children than in adults during a 5 year-survey (January 2010 to December 2014) among all patients with *Salmonella* infection admitted to the emergency room at the University Hospital in Pointe-à-Pitre (31 cases versus one in a 87-year-old woman). In addition, no asymptomatic carriers or secondary cases were identified (unpublished data). Reptile-related salmonellosis is also known to lead to invasive disease in young age [9,10]. All these elements suggest that serovars Panama and Arechavaleta are probably poorly adapted to humans.

In conclusion, the data reported here add to understanding of the epidemiology of *Salmonella* in Guadeloupe and, by extension, in the Caribbean. Panama and Arechavaleta were the two serovars most often recovered in humans. Their presence in wild reptiles and pigs suggests a broad host range and that human infections may result from indirect or direct contact with animals.

## Supporting information

S1 Table Details of GPS coordinates of sampling locations of cold-blooded animals, number of positive animals and isolated serovars. (XLSX)
